# Targeting Autophagy Using Long Non-Coding RNAs (LncRNAs): New Landscapes in the Arena of Cancer Therapeutics

**DOI:** 10.3390/cells12050810

**Published:** 2023-03-06

**Authors:** Aviral Kumar, Sosmitha Girisa, Mohammed S. Alqahtani, Mohamed Abbas, Mangala Hegde, Gautam Sethi, Ajaikumar B. Kunnumakkara

**Affiliations:** 1 Cancer Biology Laboratory, Department of Biosciences and Bioengineering, Indian Institute of Technology (IIT) Guwahati, Guwahati 781039, Assam, India; 2 Radiological Sciences Department, College of Applied Medical Sciences, King Khalid University, Abha 61421, Saudi Arabia; 3 BioImaging Unit, Space Research Centre, Michael Atiyah Building, University of Leicester, Leicester LE1 7RH, UK; 4 Electrical Engineering Department, College of Engineering, King Khalid University, Abha 61421, Saudi Arabia; 5 Electronics and Communications Department, College of Engineering, Delta University for Science and Technology, Gamasa 35712, Egypt; 6 Department of Pharmacology, Yong Loo Lin School of Medicine, National University of Singapore, Singapore 117600, Singapore; 7 NUS Center for Cancer Research (N2CR), Yong Loo Lin School of Medicine, National University of Singapore, Singapore 117600, Singapore

**Keywords:** lncRNAs, autophagy, cancer, therapeutics

## Abstract

Cancer has become a global health hazard accounting for 10 million deaths in the year 2020. Although different treatment approaches have increased patient overall survival, treatment for advanced stages still suffers from poor clinical outcomes. The ever-increasing prevalence of cancer has led to a reanalysis of cellular and molecular events in the hope to identify and develop a cure for this multigenic disease. Autophagy, an evolutionary conserved catabolic process, eliminates protein aggregates and damaged organelles to maintain cellular homeostasis. Accumulating evidence has implicated the deregulation of autophagic pathways to be associated with various hallmarks of cancer. Autophagy exhibits both tumor-promoting and suppressive effects based on the tumor stage and grades. Majorly, it maintains the cancer microenvironment homeostasis by promoting viability and nutrient recycling under hypoxic and nutrient-deprived conditions. Recent investigations have discovered long non-coding RNAs (lncRNAs) as master regulators of autophagic gene expression. lncRNAs, by sequestering autophagy-related microRNAs, have been known to modulate various hallmarks of cancer, such as survival, proliferation, EMT, migration, invasion, angiogenesis, and metastasis. This review delineates the mechanistic role of various lncRNAs involved in modulating autophagy and their related proteins in different cancers.

## 1. Introduction

Cancer has been hailed as one of the deadliest diseases of the 21st century, with a significant impact on patients’ economics and quality of life [1]. Different treatment regimens are available in the battle against cancer, but their effect is limited based on the tumor grade, stage, and type [2,3]. Novel methods have tried to determine the possible targets for early detection, but still, much research is required to firmly establish these targets to be used as effective prognostic and diagnostic tools. With the identification of several types of cancers, their mechanisms, pathways involved, and their causes, it has become even more imperative to identify further the causative factors that could help diagnose and combat the disease [4,5,6]. The shift towards more personalized approaches for the treatment of this disease has led to an increase in the overall survival of patients [7].

Autophagy is an evolutionary conserved, multi-step, biological process of cellular degradation and elimination of damaged or misfolded proteins and cellular organelles that occurs in response to stressful stimuli [8,9]. The word autophagy (from the Greek term “auto”, meaning oneself, and “phagy”, referring to eat) was first coined by Nobel laureate Christian de Duve in 1963 at the Ciba Foundation Symposium on Lysosomes in London [10]. Although many researchers have tried to delve into the molecular mechanism governing autophagy, only recently, in 2016, Yoshinori Ohsumi was awarded the Nobel Prize for Physiology or Medicine for his research on the mechanism of autophagy and its effect on human health and disease [11]. Autophagy helps to maintain cellular homeostasis by engulfing the aggregates or organelles in membrane vesicles, which are then transported to the lysosome for degradation [12]. Moreover, the basal activation of autophagy plays a vital role in the maintenance of organelle quality control [13]. It is also established that autophagy plays a crucial role in immune surveillance, as peptides of pathogens degraded by autophagic pathways can present antigens to immune cells, thereby regulating host defense and immunity [14,15,16]. In the absence of stress stimuli, such as hypoxia, nutrient deprivation, the presence of pathogens, and the accumulation of misfolded proteins, autophagy is active at basal levels to recycle the nutrients and maintain the energetics of the cell [17]. However, autophagy is upregulated in response to various stresses to preserve cellular homeostasis. Autophagy can be stratified into three major types depending upon the route of cargo delivery to the lysosome, which are macroautophagy, microautophagy, and chaperon-mediated autophagy (CMA) [18,19]. Macroautophagy is the most widely studied mechanism, wherein the formation of autophagosomes and autolysosomes takes place. The autophagosomes, double membrane vesicles, engulf the protein aggregates and (a portion of) organelles, and fuse with the lysosome to form autolysosomes [20]. Microautophagy, a process of cell eating, involves the direct engulfment of cytoplasmic contents into the lysozymes at the membrane boundary by autophagic tubes. The major functions of microautophagy in cellular homeostasis are the maintenance of organelle size, membrane dynamics, and survival under nitrogen restrictions [21,22]. In CMA, cytosolic proteins with the pentapeptide sequence KFERQ are identified by the heat shock cognate (HSC70; also known as HSPA8) to form a complex. LAMP2A or the lysosomal-associated membrane protein 2A facilitates the translocation of the chaperone complex into the lysosome [23]. The various autophagy processes are strictly regulated by a set of autophagy-related genes (*ATG*s) [24,25].

A plethora of studies have elucidated the crucial involvement of autophagy in various human disorders, including cancer [26,27]. As autophagy is a catabolic degradative process, it prevents the accumulation of cellular damage that could otherwise lead to cancer initiation. Autophagy acts as a double-edged sword in cancer development and progression by playing a multi-faceted role in both tumor-promoting and suppressing functions [12,28]. In the early stages of cancer, autophagy has tumor-suppressive functions through the degradation of harmful and damaged proteins/organelles, thus minimizing the accumulation and spread of damage. However, in the advanced stages of the disease, it exhibits tumor-promoting effects by maintaining the vitality and viability of the tumor under the stress microenvironment [29]. As tumors require sustained proliferation, nutrient demand is crucial for their progression. Metabolic reprogramming by the tumor activates autophagy to recycle the nutrients and channel the energy back to the tumor [30]. Initial evidence for a possible direct connection between autophagy and cancer came from the discovery of Beclin-1 as a haploinsufficient tumor suppressor with mutations in cancers of the ovary, small intestines, and skin [31]. Moreover, it has been reported that the activation of RAS, a well-known oncogene, induces autophagy through signaling pathways, such as Raf-1/ERK, PI3K/mTOR, and Rac1/JNK [32]. Various studies have linked autophagy to be involved in the maintenance of cancer dormancy [33,34,35,36]. Cellular dormancy refers to a halt in the proliferative abilities of the cells, where they enter into a quiescent-like state and rest in the G0–G1 phase of the cell cycle. The dormant cancer cells utilize autophagy to survive in hypoxic and nutrient-deficit conditions in the tumor microenvironment [33]. These dormant cells may reside in the tumor for a long time and not respond to therapies, leading to metastasis and disease recurrence [37]. Additionally, autophagy is thought to contribute to tumor cells’ lower vulnerability to NK cell eradication. According to Baginska et al., hypoxia-induced autophagy prevented the NK cell-mediated destruction of MCF-7 breast cancer cells [24]. The deprivation of glucose and amino acids triggers HIF-1-independent autophagy through AMPK activation and mTOR inhibition [38]. Autophagy can be employed with immunosurveillance for the non-cellular autonomous prevention of cancer. For instance, reduced autophagy is linked to regulatory T-cell infiltration, which suppresses the immune system and reduces the effectiveness of immunosurveillance, thus increasing tumor development [39]. Therefore, increasing autophagy in premalignant lesions might be a plausible approach for inhibiting tumor progression. Recently, there have been different clinical interventions specifically modulating or targeting autophagy in cancer therapy, with the vast majority focusing on inhibiting autophagy [12,30]. Thus, understanding the intricate molecular regulation of autophagy and its distinct functions is essential for the development of cutting-edge cancer treatments.

Recently, there has been increased attention towards non-coding RNAs (ncRNAs) and their role in regulating various biological processes, such as survival, proliferation, differentiation, apoptosis, the immune response, metabolism, and homeostasis [40,41,42,43]. Among various ncRNAs, long non-coding RNAs (lncRNAs) have emerged as important players in modulating diverse molecular and biological functions [44]. lncRNAs are ncRNAs of more than 200 nucleotides in length and are involved in transcriptional and post-transcriptional gene regulation [45,46]. Like most non-coding RNAs, lncRNAs are transcribed by RNA polymerase II, containing a 5′ methyl-cytosine cap and 3′ poly(A) at the tail [47]. Various mechanisms are associated with the formation of lncRNAs, such as the generation of mature end by ribonuclease P (RNaseP), cleavage, and the formation of complex caps from small nucleolar RNA (snoRNA) and protein (snoRNP) [48,49]. In one study, sub-nuclear structures called “paraspeckles” were found to be present in the biogenesis of specific lncRNA [50]. Still, the exact mechanism of lncRNA synthesis remains unclear, and more in-depth studies are vital in understanding the functional role of lncRNAs in gene regulation. lncRNAs can act as scaffolds, decoys, enhancers, and guides to bind and regulate DNA, RNA, and proteins [51]. Further, lncRNAs can behave as competing endogenous RNAs (ceRNAs), which sequester the microRNAs (miRNA) by competing with and sharing similar miRNA-response elements, thus regulating the expression of miRNAs and the gene [52]. As the research on lncRNAs is constantly evolving, the functional classification of lncRNA is not present to date. Recent studies have linked lncRNA deregulation with various pathophysiological processes of different human disorders, including cancer [53,54,55,56]. lncRNAs can act as oncogenes and tumor suppressors, thereby modulating different hallmarks of cancer, such as survival, proliferation, apoptosis, migration, invasion, epithelial-to-mesenchymal transition (EMT), angiogenesis, and metastasis [57,58,59,60,61]. There have been reports in which lncRNAs regulate autophagy by modulating the expression of various *ATG* genes. Majorly, lncRNAs act as ceRNAs to sequester miRNAs targeting the autophagic process [62,63]. This review focuses on the functional role of different lncRNAs modulating autophagy in various cancers. Further, it provides insights into the autophagy-related lncRNAs and their regulation of *ATG* genes in defining the autophagic process of initiation, phagophore formation, autophagosome elongation/closure, and autolysosome fusion. Targeting autophagy through lncRNAs would be a novel treatment regimen in circumventing this deadly malady.

## 2. LncRNAs as Mediators of the Autophagy Process

**Figure 1:**
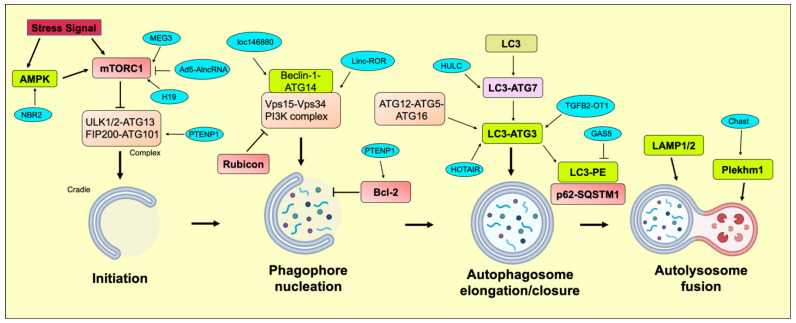
Mechanistic action of different lncRNAs in regulating the autophagic process of initiation, phagophore formation, autophagosome elongation/closure, and autolysosome fusion.

Autophagy is a multi-step and dynamic process that eliminates accumulated misfolded proteins or damaged organelles. Although there are three major types of autophagy, macroautophagy has been extensively studied and well-researched [62]. Here, we have discussed the major phases of autophagy with an emphasis on lncRNAs as regulators of different *ATG*-related genes (Figure 1).

### 2.1. Initiation

Two major classes of sensing proteins that are involved in autophagy initiation are the mammalian target of rapamycin complex 1 (mTORC1) and adenine monophosphate-activated protein kinase (AMPK) [64,65,66]. During nutritional starvation, the phosphorylation of AMPK leads to the inhibition of mTORC1, which induces the ATG1/ULK1/2 complex to initiate the autophagic process [64,66]. In addition, it has been well-reported that a PI3K/AKT/mTOR signaling cascade is negatively associated with the initiation of autophagy [67]. Then, ATG17 forms a complex with ATG31 and ATG29 and other autophagy-related genes, ATG13 and ATG1, to form the pre-autophagosomal structure (PAS) in yeast systems [68,69]. A stable complex is formed in mammalian cells with the help of the ULK1/2 and ATG101 complex in conjugation with ATG13 and FIP200/RB1CC1, which is then transferred to omegasomes (the site of autophagosome synthesis) [69,70].

Zhou and his colleagues elucidated the functional role of lncRNA H19 in autophagy initiation. It was reported that high glucose levels decreased H19 expression leading to transcriptional inactivation of DIRAS3 via inhibition of the PI3K/AKT/mTOR signaling cascade. Further, the knockdown of H19 increased ATG7 and Beclin-1 levels, indicating a link between the H19 and major autophagy-related genes [71]. In another study, it was observed that lncRNA H19 activated autophagy via the repression of DUSP5, a type of mitogen-activated kinase phosphatase. It is well known that DUSP5 inhibits ERK1/2 phosphorylation, which can suppress autophagy [72]. Recently, a study reported that lncRNA neighbor of BRCA1 gene 2 (NBR2) could induce the activation of AMPK through direct binding. Moreover, the expression of NRB2 was found to be regulated by increasing AMPK activation under stress stimuli [73]. Furthermore, another study demonstrated the link between miR-19a and NRB2 in acute liver failure. It was observed that miR-19a suppresses autophagy by regulating the NBR2/AMPK axis [74]. It has been found that upregulating the artificial lncRNA Ad5-AlncRNA resulted in sponging multiple miRNAs (miR-216a, miR-21, miR-494, and miR-217), which targeted PTEN. This led to an increase in PTEN levels, inhibiting the AKT/mTOR pathway, thus activating autophagy in hepatocellular carcinoma (HCC) [75]. Studies have suggested that the knockdown of lncRNA HOXA transcript antisense RNA myeloid-specific 1 (HOTARIM1) results in the inhibition of autophagy and suppression of all-trans retinoic acid (ATRA)-induced degradation of RARA in promyelocytic leukemia cells [76,77]. It was observed that lncRNA maternally expressed gene 3 (MEG3) overexpression induces autophagy by directly binding to the ATG3 protein, thus preventing its degradation in ovarian carcinoma and inhibiting tumorigenesis [78]. Interestingly, lncRNA PTEN pseudogene-1 (PTENP1) has a similar 3′ UTR to that of PTEN, a tumor suppressor gene, and hence, it confers a protective role to PTEN from its candidate miRNAs [79]. The overexpression of PTENP1 results in increased levels of PTEN, leading to repression of the PI3K/Akt pathway and the activation of autophagy. Moreover, it has been observed that PTENP1 can sequester miR-20a and miR-17 to increase the expression of ATG7, ULK1, and p62/SQSTM1 [80].

### 2.2. Phagophore Nucleation

After activation of the omegasome/PAS, the ATG1/ULK1 complex induces the PI3K complex, consisting of Vps15, Vps34, Beclin 1, and Barkor, to form phosphatidylinositol 3-phosphate (PI3P) [17,81,82]. PI3P promotes formation of the omegasome through the recruitment of double FYVE-containing protein 1 (DFCP1) [83]. In addition, various regulators of autophagy, such as VMP1 and ATG9, are active in the autophagic membrane [69,84]. It has been found that Bcl-2 and Rubicon are negative regulators of phagophore nucleation by modulating the formation of the class III PI3K complex [85,86].

The inhibiting long intergenic non-protein coding RNA, regulator of reprogramming (linc-ROR) can induce autophagy by increasing Beclin-1 expression and inducing gemcitabine and tamoxifen resistance in breast cancer. However, more research is required to determine how linc-ROR modulates the expression of Beclin-1 and whether linc-ROR could be used in clinical settings [87,88]. LncRNA loc146880 has been found to be closely associated with lung cancer pathogenesis and autophagy. Induction with PM2.5 (a particulate matter) results in high levels of reactive oxygen species (ROS), and it increases the expression of the lncRNA loc146880, which activates autophagy and consequently promotes lung cancer cell invasion and migration [89]. Treatment with cisplatin increases the expression of lncRNA AC023115.3 and facilitates cisplatin-induced apoptosis. Additional mechanistic investigations have shown that lncRNA AC023115.3 sponges miR-26a, thereby increasing glycogen synthase kinase-3 (GSK3) expression [90]. Together, linc-ROR and loc146880 can influence vesicle nucleation by regulating Beclin-1 gene or protein expression.

### 2.3. Autophagosome Elongation/Closure

Ubiquitin-like conjugation systems have an important function in the autophagosome elongation/closure of the membrane. ATG12 conjugates to ATG5 and subsequently interacts with ATG16 to form the ATG12–ATG5–ATG16 complex under the control of ATG7 (E1-like enzyme) and ATG10 (E2-like enzyme) [91,92]. Then, the newly formed ATG5–ATG12–ATG16 complex helps LC3B (ATG8) to transform into its soluble cytosolic form (LC3-I) to the membrane-anchored LC3-II [93]. The neighbor of BRCA1 gene 1 (NBR1), Nix, p62, and other adaptor proteins, such as ATG19 and ATG32, selectively mediate the degradation of proteins or organelles by attracting them to autophagosomes via binding to LC3-II [93].

Research on lncRNA TGFB2 overlapping transcript 1 (TGFB2-OT1) has revealed that vascular endothelial inflammation triggers the upregulation of TGFB2-OT1, which in turn increases the expression of ATG7, ATG3, and p62 expression, plausibly by increasing the LARP1 levels and sponging miR-4459 [94]. In another study, the overexpression of GAS5 resulted in decreased levels of ATG5, ATG7, Beclin-1, ATG3, ATG12, and LC3B expression, thereby repressing autophagy [95]. Another lncRNA, prostate cancer gene expression marker 1 (PCGEM1), promotes autophagy by increasing the mRNA levels of ATG3, ATG5, ATG12, and Beclin-1 [96]. It was found that lncRNA HNF1A-AS1 can prevent miR-30b-5p from interacting with its target ATG5 and thus repress autophagy in hepatocellular cancer (HCC) [97]. In another study, lncRNA HOX antisense intergenic RNA (HOTAIR) expression was upregulated in HCC and promoted proliferation by increasing ATG3 and ATG7. Since many miRNAs, including miR-34a, miR-331-3P, miR-130a, and miR-454-3p, can interact with HOTAIR, it can control autophagy in two different ways. Firstly, to prevent miRNA transcription, HOTAIR may work as a scaffold to draw in epigenetic modification enzymes. Secondly, HOTAIR may act as a sponge to capture miRNAs from their targets [98].

### 2.4. Autolysosome Fusion

The last step of the autophagic process is the fusion of lysosomes to the autophagosomes to form an autolysosome, wherein the final degradation takes place [93]. The Rab–SNARE system and the lysosome membrane proteins LAMP1 and LAMP2 are the key molecules involved in autolysosome fusion [99,100]. Further, various adaptor proteins are crucial to connect the lysosome to the endocytic and autophagic processes. One such adaptor protein, known as pleckstrin homology domain-containing protein family M member 1 (Plekhm1), mediates the fusion of endosomes and autophagosomes with lysosomes by directly associating with the homotypic fusion and protein sorting complex and possessing an LC3-interacting region [101]. LncRNA cardiac hypertrophy-associated transcript (Chast) has been found to repress autophagy by decreasing the Plekhm1 levels and possibly ATG5 expression. This Chast/Plekhm1 axis regulates the fusion of autophagosomes to the lysosomes [102].

## 3. LncRNAs Targeting Autophagy in Different Cancers

**Figure 2:**
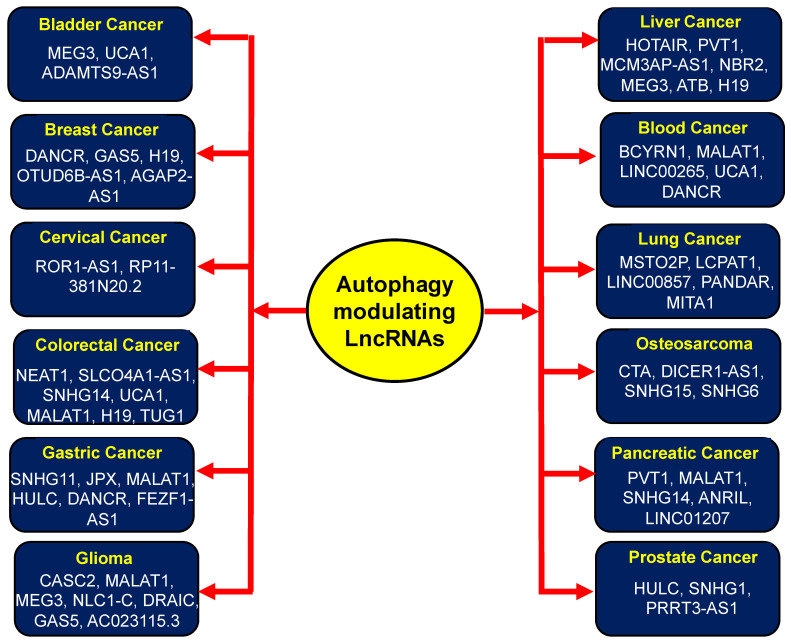
Autophagy-modulating lncRNAs targeting various types of cancers.

Accumulating evidence has implicated various lncRNAs regulating autophagy in different types of cancer [103,104,105,106,107,108,109,110]. In most cases, lncRNAs work as competing endogenous RNAs (ceRNAs) by sequestering the autophagy-related miRNAs, thereby regulating the ATG genes responsible for autophagy [53]. Recent investigations have elucidated the mechanistic role of different lncRNAs involved in autophagic regulation in tumorigenesis (Table 1). Herein, various lncRNAs that have been found to target the autophagy process have been described in cancers of the bladder, breast, cervical colon, lung, liver, blood, bone, brain, pancreas, and prostate, etc. (Figure 2; Table 2).

### 3.1. Bladder Cancer

Bladder cancer originates in the lining of the bladder, and it accounts for 212,536 cancer-related deaths worldwide [1]. Although it is a rare type of malignancy, recent statistics show its increasing prevalence worldwide with 573,278 cases reported in 2020 [1,111,112]. Very few lncRNAs have been found to play a vital role in regulating autophagy in bladder cancer [113]. Ying and their colleagues elucidated the functional role of lncRNA MEG3 in bladder tumorigenesis. LncRNA MEG3 was found to be downregulated in the bladder tumor tissues compared to levels in the normal tissue samples. It was also found that MEG3 was negatively correlated with levels of the autophagic marker LC3II; therefore, the knockdown of MEG3 activated autophagy by regulating LC3II levels. Moreover, MEG3 silencing inhibited apoptosis and induced T24 cell proliferation [114]. Another study reported that lncRNA urothelial carcinoma-associated 1 (UCA1) was involved in regulating ATG7 levels by sponging miR-582-5p. In addition, in vivo studies demonstrated that UCA1 was involved in bladder cancer progression, and the knockdown of UCA1 inhibited growth, migration, and invasion in bladder cancer cells [115]. Further, lncRNA ADAMTS9-AS1 was shown to regulate apoptosis and autophagy in bladder cancer. Silencing ADAMTS9-AS1 was found to induce autophagy and apoptosis by upregulating the LC3-II/I ratio, Beclin-1, Bax, and Caspase 9 levels while decreasing the expression of p62 and Bcl-2 in T24 and 5637 bladder cancer cell lines [116]. Hence, lncRNAs, such as MEG3, UCA1, and ADAMTS9-AS1, have been shown to regulate autophagy in bladder cancer; however, further research is crucial to elucidate their effects and mechanism in bladder carcinogenesis.

### 3.2. Breast Cancer

Breast cancer is the most common malignancy among women and accounts for the highest number of incidences reported in the year 2020 worldwide [1,117,118]. Recent studies have found that lncRNAs are crucial regulators of autophagy in breast cancer [119,120,121]. A study conducted by Zhang and group showed that the knockdown of lncRNA differentiation antagonizing non-protein coding RNA (DANCR) promoted autophagy and apoptosis and inhibited breast cancer cell proliferation by increasing Caspase 3 and 9, Atg5, LC3B, and Bax/Bcl-2 while decreasing Beclin-1. Moreover, it was revealed that DANCR acts as a competitive endogenous RNA sponge to regulate the expression of PAX6 by targeting miR-758-3p [120]. LncRNA HOTAIR was also found to be an important regulator of autophagy in breast cancer. It was observed that there are many different mechanisms of interplay between autophagy and HOTAIR in breast cancer; however, further research is required to find the underlying mechanisms [122]. Another study revealed that lncRNA growth arrest-specific transcript 5 (GAS5) is a significant contributor to the pathogenesis of breast cancer. The overexpression of GAS5 led to an increase in LC3B and Beclin-1 expression and chemosenstivity towards cisplatin. This was mainly achieved by promoting autophagy in breast cancer cells through the regulation of ULK1/ULK2 [121]. Another study observed that H19 was overexpressed in tamoxifen-resistant breast cancer cells, and the knockdown of lncRNA H19 abrogated autophagy by decreasing LC3-II and Beclin-1 levels. Interestingly, the overexpression of H19 in MCF7 tamoxifen-sensitive cells could reiterate tamoxifen resistance [119]. Other studies have also demonstrated the functional role of different lncRNAs in regulating autophagy in breast cancer [123,124]. Hence, lncRNAs, such as DANCR, HOTAIR, H19, and GAS5, are identified as key regulators of autophagy in breast cancer, and further research needs to be carried out to identify their potential in breast cancer diagnosis and treatment.

**Table 1:** Different autophagy-modulating lncRNAs and their expression in various cancers.

Cancer	LncRNAs	Clinical/In Vitro	Model	Expression	References
Acute myeloid leukemia	LINC00265	Clinical	Peripheral venous blood	Up	[125]
		In vitro	OCI/AML-2, and THP-1	Up	
	DANCR	In vitro	Ara C-treated primary cell lines and HL60, U937, and KG1a	Up	[126]
Breast Cancer	DANCR	Clinical	Breast cancer tissues	Up	[120]
		In vitro	HCC1937, 1590, ZR-75-30, and MDA-MB-468	Up	
	GAS5	Clinical	Breast cancer tissues	Down	[121]
		In vitro	MCF-7 and MDA-MB-231	Down	
	H19	Clinical	Tamoxifen-resistant breast cancer tissues	Up	[119]
		In vitro	MCF7/TAMR	Up	
	AGAP2-AS1	In vitro	Exosomes from SKBR-3-TR	Up	[124]
Bladder Cancer	MEG3	Clinical	Bladder cancer tissues	Down	[114]
	UCA1	Clinical	Bladder cancer tissues	Up	[115]
		In vitro	HT-1376, T24, J82, 5637, and EJ	Up	
	ADAMTS9-AS1	In vitro	J82, EJ, 5637, and T24	Up	[116]
Cervical Cancer	ROR1-AS1	Clinical	Cervical cancer tissues	Up	[127]
		In vitro	SiHa, ME-180 and SW756	Up	
	RP11-381N20.2	Clinical	Cervical cancer tissues and chemotherapy insensitive cervical cancer tissues	Down	[128]
Clear Cell Renal Cell Carcinoma	TUG1	Clinical	Clear cell renal cell carcinoma tissues	Up	[129]
		In vitro	786-0 and A498	Up	
Colon Cancer	EGOT	Clinical	Colon cancer tissues and serum	Up	[130]
	CASC2	Clinical	Colon cancer tissues	Down	[131]
		In vitro	HT-29, SW948, RKO, and SW480	Down	
	LINC00858	Clinical	Colon cancer tissues	Up	[132]
		In vitro	CT26, SW480, HCT116, and SW620	Up	
	KCNQ1OT1	Clinical	Colon cancer tissues	Up	[133]
Colorectal Cancer	NEAT1	Clinical	Colon cancer tissues	Up	[134]
		In vitro	HT29, HCT8, HCT116, SW480, and SW620	Up	
	SLCO4A1-AS1	Clinical	Colon cancer tissues	Up	[135]
		In vitro	SW620, SW480, HT29, DLD-1, and RKO	Up	
	SNHG14	Clinical	Colon cancer tissues	Up	[136]
		In vitro	SW620 and SW480	Up	
	UCA1	Clinical	5-FU resistant-CRC tissues	Up	[137]
		In vitro	SW480/5-FU and SW620/5-FU	Up	
	MALAT1	Clinical	Colorectal cancer tissues	Up	[138]
		In vitro	HCT290, HCT116, SW480, and SW620	Up	
	CPS1-IT1	Clinical	Colorectal cancerous tissues	Down	[139]
		In vitro	LoVo, SW620, SW480, and LS174T	Down	
	H19	Clinical	Colorectal cancer tissue	Up	[140]
		In vitro	5-Fu resistant SW1116 and acquired 5-Fu resistant HCT8Fu	Up	
	CASC9	In vitro	HT-29, SW480, and HCT-116	Up	[141]
	SNHG8	In vitro	HCT116, HCT8, HT29, and SW480	Up	[109]
	TUG1	Clinical	Colorectal cancer tissue	Up	[142]
		In vitro	HT-29, DLD-1, LS513, LoVo, and HCT15	Up	
Gastric Cancer	SNHG11	Clinical	Gastric cancer tissues	Up	[143]
		In vitro	AGS, BGC-823, HGC-27, MGC-803, SGC-7901, and MKN45	Up	
	JPX	Clinical	Gastric cancer tissues	Up	[144]
		In vitro	NCI-N87 and MKN45	Up	
	LINC01572	Clinical	DDP-Resistant gastric cancer tissues	Up	[145]
		In vitro	BGC823/DDP and SGC7901/DDP	Up	
	CRNDE	In vitro	Oxaliplatin and 5-FU resistant MGC-803	Down	[146]
	MALAT1	Clinical	Gastric cancer tissues	Up	[147]
	MALAT1	In vitro	SGC7901/VCR	Up	[148]
	HAGLROS	Clinical	Gastric cancer tissues	Up	[149]
		In vitro	SGC-7901, BGC-823, HGC-27, MGC-803, and AGS	Up	
	MALAT1	In vitro	CDDP-resistant GC cell lines (AGS/CDDP and HGC-27/CDDP)	Up	[150]
	HULC	In vitro	SGC7901/CDDP and MGC-803/CDDP cells transfected with LV-METase	Down	[151]
	EIF3J-DT	In vitro	MGC-803/OXA and MGC-803/5Fu	Up	[152]
	DANCR	Clinical	Gastric cancer tissues	Up	[153]
		In vitro	SGC7901, MGC-803, and NCI-N87	Up	
	CCAT1	In vitro	AGS, and MKN-45	Up	[154]
	LIT3527	Clinical	Gastric cancer tissues	Up	[155]
		In vitro	AGS, MKN45, MKN74, SGC7901, and MGC-803	Up	
	FEZF1-AS1	Clinical	Gastric cancer tissues	Up	[108]
		In vitro	MKN-49P, MGC-803, BGC-823, SGC-7901, and NCI-N87	Up	
	LINC00963	Clinical	Gastric cancer tissues	Up	[156]
		In vitro	SGC-7901, MKN45, and MKN74	Up	
Glioma	MALAT1	Clinical	Glioma tissues	Up	[105]
		In vitro	U87, U118, U251, U373 and D247	Up	
	CASC2	Clinical	Glioma and peritumoral brain edema (PTBE) tissues	Down	[157]
		In vitro	Temozolomide resistance U257 and U87	Down	
	MEG3	Clinical	Glioma tissues	Down	[158]
		In vitro	U251	Down	
	GAS5	In vitro	U87, U251, U138 and LN18	Down	[159]
	AC023115.3	In vitro	Cisplatin-induced U87MG	Up	[90]
	Linc-RA1	In vitro	Radioresistant glioma cell lines (M059K and U87)	Up	[160]
	H19	In vitro	U87, and U251	Up	[161]
	LINC00470	In vitro	Exosomes from glioma patient’s serum	Up	[162]
	Lnc-NLC1-C	In vitro	U87MG	Up	[163]
Head and Neck Squamous Cell Carcinoma	LINC00460	Clinical	Head and neck squamous cell carcinoma tissues	Up	[164]
		In vitro	PCI-13, FaDu, SCC-15, and UM-SCC-10A	Up	
Hepatocellular Cancer	SNHG11	Clinical	Hepatocellular cancer tissues	Up	[165]
		In vitro	SK-HEP-1, Hep G2, HuH-7, and Li-7	Up	
	HOTAIR	Clinical	Hepatocellular cancer tissues	Up	[98]
		In vitro	Huh7, HepG2, and BEL-7402	Up	
	PVT1	Clinical	Hepatocellular cancer tissues	Up	[166]
		In vitro	Bel-7402, Hep3B, and HepG2	Up	
	CCAT1	Clinical	Hepatocellular cancer tissues	Up	[167]
		In vitro	Huh7, HCCLM3, Hep3B, and HepG2	Up	
	HNF1A-AS1	Clinical	Hepatocellular cancer tissues	Up	[97]
		In vitro	HepG2, SMMC-7721, and Huh7	Up	
	NBR2	Clinical	Hepatocellular cancer tissues	Down	[168]
		In vitro	HepG2, PLC/PRF/5, Hep3B, Huh7, MHCC-97 L, MHCC-97H, SK-Hep1, and MHCC-LM3	Down	
	NEAT1	In vitro	HepG2, Huh7, Hep3B, and SMMC-7721	Up	[169]
	H19	In vitro	Hypoxia/reoxygenation (h/R)-induced HepG2 and HCCLM3	Up	[170]
	DCST1-AS1	Clinical	Hepatocellular cancer tissues	Up	[171]
	HAGLROS	Clinical	Hepatocellular cancer tissues	Up	[172]
		In vitro	SK-Hep1, MHCC97L, MHCC97H, Huh7, and HepG2.2.15	Up	
	DANCR	Clinical	Hepatocellular cancer tissues	Up	[173]
	HULC	Clinical	Hepatocellular cancer tissues	Up	[174]
	ATB	Clinical	Hepatocellular cancer tissues	Up	[175]
	CRNDE	Clinical	Hepatocellular cancer tissues	Up	[103]
		In vitro	SMMC-7721, HepG2, Hep3B, Huh7, and PLC	Up	
	RP11-295G20.2	Clinical	Hepatocellular cancer tissues	Up	[176]
	CCAT2	Clinical	Hepatocellular cancer tissues	Up	[177]
	HnRNPU-AS1	Clinical	Hepatocellular cancer tissues	Down	[178]
Laryngeal Squamous Cell Carcinoma	H19	Clinical	Laryngeal squamous cell carcinoma tissues	Up	[179]
		In vitro	TU-177/R and AMC-HN-8/R	Up	
Lung Cancer	MSTO2P	Clinical	Lung cancer tissues	Up	[180]
		In vitro	H1299, H23, and A549	Up	
	LCPAT1	In vitro	CSE- or PM2.5-induced H1299 and H520	Up	[181]
	PANDAR	Clinical	Lung cancer tissues	Down	[182]
		In vitro	L78, PC9, 95D, NCI-H460, and A549	Down	
	MITA1	In vitro	Gefitinib-resistant HCC827GR	Up	[110]
Lymphoma	BCYRN1	Clinical	Extranodal NK/T-cell lymphoma samples	Up	[183]
Multiple Myeloma	MALAT1	Clinical	Bone marrow mononuclear cells	Up	[106]
		In vitro	KM3 and U266	Up	
Nasopharyngeal Cancer	MEG3	Clinical	Nasopharyngeal cancer tissues	Down	[184]
		In vitro	C666-1, HK-1, 5-8F, and 6-10B	Down	
Non-Small Cell Lung Cancer	NBAT1	Clinical	Non-small cell lung cancer tissues	Down	[185]
	BLACAT1	In vitro	A549/DDP and H1299/DDP	Up	[186]
	GAS5	Clinical	Non-small cell lung cancer tissues	Down	[187]
	PVT1	Clinical	Non-small cell lung cancer and cisplatin-resistant tissues	Up	[188]
		In vitro	A549 and A549/DDP	Up	
Osteosarcoma	CTA	Clinical	Osteosarcoma tissues	Down	[189]
		In vitro	DOX-resistant MG-63	Down	
	DICER1-AS1	In vitro	MG-63, U2OS, HOS, 143B, and Saos-2	Up	[107]
	SNHG15	Clinical	Osteosarcoma tissues	Up	[190]
		In vitro	143B, U2OS, HOS, MG63, and Saos-2	Up	
	SNHG6	Clinical	Osteosarcoma tissues	Up	[191]
		In vitro	SOSP-9607 and MG63	Up	
Ovarian Cancer	HOXA11-AS	In vitro	SKOV3, OVCAR3, and A2780	Up	[192]
	TUG1	Clinical	Ovarian cancer tissues	Up	[193]
		In vitro	A2780, A2780/R, and SKOV3	Up	
	XIST	In vitro	SKOV3, A2780, and HO-8910	Up	[194]
Pancreatic Cancer	LINC01207	Clinical	Pancreatic cancer tissues	Up	[195]
	PVT1	In vitro	Gemcitabine-resistant PANC-1 and SW1990	Up	[196]
	MALAT1	Clinical	Pancreatic ductal adenocarcinoma tissues	Up	[197]
		In vitro	CFPAC, Bxpc-3, and PANC-1	Up	
	SNHG14	In vitro	SW1990	Up	[198]
	ANRIL	Clinical	Pancreatic cancer tissues	Up	[199]
		In vitro	PANC-1, ASPC-1, HPAC, and BxPC-3	Up	
Papillary Thyroid Cancer	BANCR	Clinical	Papillary thyroid cancer tissues	Up	[200]
		In vitro	IHH-4	Up	
Prostate Cancer	SNHG1	Clinical	Prostate cancer tissues	Up	[201]
		In vitro	LNCaP, PC-3, and DU-145	Up	
	PRRT3-AS1	In vitro	PC3, DU145, LNCaP, IA8, and IF11	Up	[104]
Retinoblastoma	MALAT1	In vitro	Y79, Weri-Rb1, SO-Rb50, and HXO-RB44	Up	[202]

### 3.3. Cervical Cancer

Cervical cancer is a malignant tumor that originates in the cervix cells and is one of the most common cancers occurring in women worldwide [203,204]. According to GLOBOCAN 2020, approximately 604,127 incidences were reported for cervical cancer [1]. Few studies have investigated the functional role of lncRNAs in modulating autophagy in cervical cancer. In line with this, Zhang and his group studied the role and effect of lncRNA ROR1 antisense RNA 1 (ROR1-AS1) in cervical cancer. It was observed that the silencing of ROR1-AS1 resulted in decreased proliferation, migration, invasion, and autophagy in cervical cancer. Moreover, ROR1-AS1 was found to regulate the expression of STC2 by sponging miR-670-3p [127]. In another study, lncRNA RP11-381N20.2 was significantly downregulated in the chemotherapy-insensitive cervical cancer patients compared with that in the chemotherapy-sensitive group. Moreover, treatment with RP11-381N20.2 was found to inhibit paclitaxel-induced autophagy in SiHa cells [128]. Thus, lncRNAs play a crucial role in regulating the process of autophagy in cervical cancer; however, further studies are required to understand its in-depth mechanistic role in modulating autophagy in cervical cancer.

### 3.4. Colorectal Cancer

Colorectal cancer (CRC) is the third most common cancer diagnosed and one of the major causes of cancer-related deaths worldwide [1,205]. CRC is a heterogeneous disease characterized by the gradual compilation of genetic and epigenetic changes, leading to the modification of normal colonic mucosa into invasive cancer [206,207,208,209,210]. Recent studies have shown that lncRNAs play a crucial role in regulating the autophagic mechanisms involved in intestinal tumorigenesis [131,132,134,137]. For instance, it was observed that nuclear paraspeckle assembly transcript 1 (NEAT1) lncRNA was highly expressed in CRC cells and tissues and promoted autophagy by directly targeting miR-34a levels. Moreover, NEAT1 was also found to be involved in promoting 5-FU-chemoresistance in CRC by indirectly regulating HMGB1 levels [134]. Another study reported that lncRNA small nucleolar RNA host gene 6 (SNHG6) inhibited the expression of miR-26a-5p and promoted ULK1-induced autophagy in RKO, HCT116, and HT29 cell lines. Further, it was also found to play a vital role in chemoresistance by sponging miR-26a-5p expression [211]. In addition, Wang and Jin explored the role of lncRNA SLCO4A1 antisense RNA 1 (SLCO4A1-AS1) in colorectal tumorigenesis. It was found that SLCO4A1-AS1 is an important factor in inducing the proliferation of cancer cells through the enhancement of autophagy via the miR-508-3p/PARD3 axis [135]. Additionally, it was found that lncRNA H19 is a mediator of autophagy in colorectal cancer cells. It was observed that the overexpression of H19 led to increased LC3-II levels and decreased p62 expression, which triggered the autophagy signaling pathway [140]. Further, in the research conducted on lncRNA metastasis-associated lung adenocarcinoma transcript 1 (MALAT1), it was observed that it is an activator of autophagy and was found to promote cell proliferation and inhibit apoptosis by sponging miR-101 in CRC. It was also observed that the suppressive effects of miR-101 on proliferation and autophagy in CRC cell lines were abolished in rescue experiments by MALAT1 [138]. 

Another study on lncRNA small nucleolar RNA host gene 14 (SNHG14) showed that it stimulates autophagy by regulating the miR-186/ATG14 axis. Further, this study concluded that SNHG14 could have a crucial role in the regulation of cisplatin resistance in CRC via autophagy [136]. LncRNA CPS1 intronic transcript 1 (CPS-IT1) was identified as an important suppressor of EMT and metastasis in CRC. This tumor suppressor role was achieved by inhibiting hypoxia-induced autophagy through inactivating HIF-1α [139]. Moreover, another study has revealed that lncRNA UCA1 accelerates 5-FU resistance in CRC cells by inhibiting apoptosis and facilitating autophagy. Further, miR-23b-3p was identified as a key target of UCA1 in CRC cells. It was also found that the knockdown of miR-23b-3p resulted in reversing the effects of UCA1 interference. Thus, UCA1 was identified as a critical regulator of autophagy [137]. In addition, a study showed that lncRNA KCNQ1 opposite strand/antisense transcript 1 (KCNQ1OT1) enhances autophagy in colon cancer. It was also found to be a promoter of chemoresistance by sponging miR-34a, thereby modulating the expression of Atg4B. Due to these multifaceted roles, KCNQ1OT1 could be a promising target for colon cancer therapeutics [133]. Additionally, another study showed that inhibition of lncRNA eosinophil granule ontogeny transcript (EGOT) promoted autophagy in colon cancer and significantly suppressed cell growth and metastasis [130]. In another study, lncRNA CASC2 was found to be an inducer of autophagy and apoptosis by modulating the expression of TRIM16 in colon cancer [131]. Finally, lncRNA LINC00858 was found to inhibit the autophagy, apoptosis, and senescence of colon cancer cells via the activation of methylation at the WNK2 promoter [132]. Hence, many studies have led us to conclude that lncRNAs play a vital role in regulating the autophagic mechanisms in colorectal cancer.

**Table 2:** LncRNAs and their mechanistic action in regulating autophagy in different cancers.

Cancer	LncRNA	Target miRNA/Gene	Effect after Overexpression/Knockdown	References
Acute Myeloid Leukemia	LINC00265 ^b^	miR-485-5p	↓LC3-II/LC3-I ratio, ↓Beclin-1, ↑p62, ↓IRF2, ↑Apoptosis	[125]
	UCA1 ^a^	miR-96-5p	↑ATG7, ↑Beclin-1, ↑Proliferation	[212]
	DANCR ^a^	miR-874-3p	↑ATG16L1, ↑Cytarabine resistance, ↑LC3-II, ↓SQSTM1/p62	[126]
Breast Cancer	DANCR ^b^	miR-758-3p	↑ATG5, ↑Caspase 3, ↑Caspase 9, ↑Bax, ↓Bcl-2, ↑LC3B, ↓Beclin-1, ↓PAX6	[120]
	GAS5 ^a^	-	↑LC3B, ↑Beclin-1, ↑ULK1, ↑ULK2, ↑Chemosensitivity	[121]
	H19 ^b^	-	↓Tamoxifen resistance, ↓Beclin-1, ↓LC3-II, ↑DNMT3B	[119]
	OTUD6B-AS1 ^a^	miR-26a-5p	↑LC3B-II, ↑γ-H2AX, ↓p-ATR, ↓p-ATM, ↓p-RAD51	[123]
	AGAP2-AS1 ^b^	ELAVL1	↓ATG10, ↓ATG5, ↓LC3-II, ↑p62, ↓Trastuzumab resistance	[124]
Bladder Cancer	MEG3 ^b^	-	↑LC3-II, ↓Apoptosis, ↓G0/G1 phase populations	[114]
	UCA1 ^b^	miR-582-5p	↓ATG7, ↑p62, ↑LC3-I/LC3-II ratio, ↑E- cadherin, ↓Zeb1, ↓Zeb2, ↓Twist, ↓Snail, ↓MRP1, ↓LRP, ↓GST, ↑TOPO-II	[115]
	ADAMTS9-AS1 ^b^	AMDAMT9	↑Beclin-1, ↑LC3-II/LC3-I ratio, ↑Caspase 9, ↑Bax, ↓vimentin, ↓N-cadherin, ↓Snail, ↑E-cadherin, ↓p62, ↓Bcl-2, ↓PIK3CB, ↓p-AKT, ↓p-mTOR	[116]
Cervical Cancer	ROR1-AS1 ^b^	miR-670-3p	↓Beclin 1, ↑LC3-I, ↓LC3-II, ↓Proliferation, ↑Apoptosis	[127]
	RP11-381N20.2 ^a^	-	↓Paclitaxel-induced autophagy, ↓ATG7, ↑ chemosensitivity	[128]
Clear Cell Renal Cell Carcinoma	TUG1 ^b^	miR-31-5p	↑LC3-II/LC3-I ratio, ↓p62, ↓PCNA, ↑cle-Caspase 3, ↓FLOT1	[129]
Colon Cancer	EGOT ^a^	-	↓cle-Caspase 3, ↓Bax, ↑Bcl-2, ↓Beclin-1, ↑p62, ↓LC3-II/LC3-I, ↑Proliferation, ↑Invasion	[130]
	CASC2 ^a^	miR-214	↓TRIM16, ↑Beclin-1, ↑LC3-II, ↑Bax, ↓Bcl-2, ↑cle-Caspase 3, ↓Proliferation	[131]
	LINC00858 ^b^	-	↑Beclin-1, ↑LC3Ⅱ/I, ↑Bax, ↓Bcl-2, ↑cle-Caspase 3, ↑p27	[132]
	KCNQ1OT1 ^b^	miR-34a	↓Atg4B, ↑cle-PARP, ↓LC3Ⅱ, ↑Chemosensitivity, ↓Proliferation	[133]
Colorectal Cancer	NEAT1 ^b^	miR-34a	↓ATG9A, ↓ATG4B, ↓Beclin-1, ↓LC3II/I ratio, ↑cle-Caspase 3, ↓ULK1, ↓HMGB1, ↑Chemosensitivity	[134]
	SLCO4A1-AS1 ^a^	miR-508-3p	↑Proliferation, ↓Apoptosis ↑LC3B-II, ↑PARD3	[135]
	SNHG14 ^b^	miR-186	↓Proliferation, ↓Migration, ↓Invasion, ↓ATG14, ↓LC3B, ↓Cisplatin resistance	[136]
	UCA1 ^b^	miR-23b-3p	↓LC3-II/LC3-I ratio, ↓Beclin-1, ↑p62, ↑Bax, ↑Caspase 3, ↓5-FU resistance, ↓ZNF281	[137]
	MALAT1 ^b^	miR-101	↓Proliferation, ↑cle-Caspase 3, ↓LC3-II/LC3-I ratio, ↑p62	[138]
	CPS1-IT1 ^a^	-	↓LC3-II, ↓HIF-1α, ↓Beclin-1, ↓N-cadherin, ↓Vimentin, ↑E-cadherin, ↑ZO-1	[139]
	H19 ^a^	miR-194-5p	↑Proliferation, ↑LC3-II, ↓p62, ↑SIRT1, ↑Chemoresistance	[140]
	SNHG6 ^b^	miR-26a-5p	↓Proliferation, ↑cle-Caspase 3, ↑cle- PARP, ↓p-ULK1, ↓ATG13, ↓ULK1, ↓Chemoresistance	[211]
	CASC9 ^b^	-	↓Proliferation, ↓Migration, ↑LC3B-II, ↓p62, ↓Vimentin, ↑E-cadherin, ↑p-AMPKα/AMPKα, ↓p-AKT, ↓p-mTOR	[141]
	SNHG8 ^a^	miR-588	↑Proliferation, ↑LC3-II, ↑ATG7,	[109]
	TUG1 ^a^	miR-195-5p	↑Proliferation ↑LC3II, ↑Beclin-1, ↓p53, ↓Bax, ↑Bcl-2, ↓Caspase 3, ↑HDGF, ↑DDX5, ↑β-catenin	[142]
Gastric Cancer	SNHG11 ^b^	miR-483-3p/miR-1276	↓LC3-II/LC3-I ratio, ↑p62, ↓LAMP1, ↓Twist, ↓Nanog, ↓LRG5, ↓CD133, ↓EpCAM, ↓Sox2, ↓Bcl-2, ↑Bax, ↓MMP-2, ↓MMP-7, ↑E-cadherin, ↓N-cadherin, ↓CUL4A, ↑GSK-3β, ↓β-catenin, ↑cle- PARP, ↑cle-Caspase 3, ↑cle-Caspase 6	[143]
	JPX ^b^	miR-197	↓Proliferation, ↓Migration, ↓Invasion	[144]
	LINC01572 ^b^	miR-497-5p	↓Autophagy, ↓Proliferation, ↓Migration, ↓Invasion, ↓Cisplatin resistance	[145]
	CRNDE ^a^	-	↑Apoptosis, ↓LC3-II, ↑cle- PARP, ↑cle-Caspase 3, ↓Chemoresistance	[146]
	MALAT1 ^a^	miR-204	↑Proliferation, ↑LC3B, ↑Ki67, ↑TRMP3	[147]
	MALAT1 ^b^	miR-23b-3p	↓LC3-II/LC3-I ratio, ↑p62, ↓ATG12, ↓Chemoresistance	[148]
	MALAT1 ^a^	miR-30b	↑Proliferation, ↑LC3-II, ↓p62, ↑ATG5, ↑Cisplatin resistance	[150]
	HULC ^a^	-	↑LC3-II/LC3-I, ↑Beclin-1, ↓p62, ↑FoxM1, ↑MDR1, ↑Cisplatin resistance	[151]
	HAGLROS ^b^	miR-100-5p	↑LC3-II/LC3-I, ↓p62, ↓p-mTOR, ↓mTOR, ↓p-4E-BP1, ↓Proliferation, ↓Migration, ↓Invasion	[149]
	EIF3J-DT ^b^	miR-188-3p	↓Proliferation, ↑cle-PARP, ↑cle-Caspase 3, ↓LC3-II, ↓ATG14, ↓Chemoresistance	[152]
	DANCR ^b^	miR-194	↑LC3-II/LC3-I ratio, ↑Beclin-1, ↑Apoptosis	[153]
	CCAT1 ^a^	miR-140-3p	↑Proliferation, ↑Migration, ↑Invasion, ↑LC3A/B, ↑Beclin-1, ↑ATG5, ↑ATG12	[154]
	LIT3527 ^b^	-	↓Proliferation, ↓Migration, ↑LC3-II ↑Apoptosis, ↓p-AKT, ↓p-mTOR, ↓p-ERK, ↓4EBP1, ↓Metastasis	[155]
	FEZF1-AS1 ^b^	-	↓LC3-II, ↓ATG5, ↑Bax, ↓Bcl-2, ↑cle-Caspase 3, ↓MDR1, ↓MPR1, ↓S-phase cell populations, ↓Chemoresistance	[108]
	LINC00963 ^b^	miR-4458	↓LC3-II, ↑p62, ↓Proliferation, ↓Migration	[156]
Glioblastoma	LINC00470 ^a^	miR-101	↑ELFN2, ↓Dicer, ↓LC3-II, ↓ATG7, ↓ATG3, ↓Beclin-1	[213]
Glioma	MALAT1 ^b^	miR-101-3p	↓LC3-II, ↑p62, ↓Proliferation, ↓STMN1, ↓RAB5A, ↓ATG4D	[105]
	CASC2 ^a^	miR-193a-5p	↓LC3-II, ↓Beclin-1, ↑p62, ↑mTOR, ↓Migration, ↓Invasion	[157]
	GAS5 ^a^	-	↓Proliferation, ↓LC3-II, ↑p62, ↑p-mTOR ↑Chemosenstivity	[159]
	AC023115.3 ^b^	miR-26a	↑LC3-II, ↓p62, ↓cle-Caspase 3, ↓cle- PARP, ↓Mcl1, ↓Chemoresistance	[90]
	Linc-RA1 ^a^	-	↓% DNA damage, ↓% Irradiation-induced death, ↑H2Bub1, ↓LC3B-II/I ratio, ↑p62, ↓γ-H2AX, ↑Radioresistance	[160]
	H19 ^a^	-	↑Proliferation, ↑Migration, ↓Autophagy, ↓p-mTOR, ↑p-ULK1	[161]
	LINC00470 ^a^	miR-580-3p	↓LC3-II/LC3-I, ↓Beclin-1, ↑p62, ↑Proliferation, ↓G1phase cell population, ↑p-PI3K, ↑p-mTOR, ↑p-AKT	[162]
	Lnc-NLC1-C ^b^	-	↓Proliferation, ↓Migration, ↓Invasion, ↑ROS generation, ↓LC3II/I, ↓p62, ↑ATG9, ↑Rab1, ↓PRDX-3	[163]
	DRAIC ^a^	-	↓LC3-II, ↑p62, ↓Migration, ↓Invasion, ↓p-ULK1 (S757), ↓p-S6K, ↑p-AMPK, ↑p-RPTOR, ↑p-FoxO3a	[214]
Head and Neck Squamous Cell Carcinoma	LINC00460 ^b^	miR-206	↓STC2, ↓AKT, ↓ERK, ↓p-ERK, ↓p-AKT, ↑G0/G1-phase cell arrest, ↑Bax, ↑cle-PARP, ↑cle-Caspase 3, ↑LC3-II/I ratio, ↑Beclin-1	[164]
Hepatocellular Cancer	SNHG11 ^b^	mir-184	↓AGO2, ↓Beclin-1, ↓LC3-II/I ratio, ↑cle-Caspase 3, ↓Migration, ↓Invasion	[165]
	HOTAIR ^a^	-	↑LC3-II, ↑ATG3, ↑ATG7, ↑Proliferation	[98]
	H19 ^b^	-	↓Proliferation, ↓G0/G1-phase cell population, ↑cle-Caspase 3, ↑cle-Caspase 9, ↓Bcl-2, ↑Cyt c, ↓LC3-II/1 ratio, ↓Beclin-1, ↑p62, ↑p-PI3K, ↑p-AKT, ↑p-mTOR	[170]
	PVT1 ^a^	miR-365	↑Proliferation, ↑Ki67, ↑LC3-II, ↑ATG3	[166]
	CCAT1 ^a^	miR-181a-5p	↑Proliferation, ↑LC3-II, ↓p62, ↑ATG7	[167]
	MEG3 ^a^	-	↓Proliferation, ↓Migration, ↓LC3-II/LC3-I, ↓Beclin-1, ↓ILF3, ↑p-PI3K, ↑p-AKT, ↑p-mTOR	[215]
	HNF1A-AS1 ^a^	miR-30b-5p	↑Proliferation, ↑LC3BII/I, ↓p62, ↑ATG5, ↑Bcl-2	[97]
	MCM3AP-AS1 ^b^	miR-455	↓Migration, ↓Vessel formation	[216]
	NBR2 ^a^	-	↓Proliferation, ↓Migration, ↓Invasion, ↓LC3 II/I ratio, ↓Beclin-1, ↑p62, ↓p-ERK, ↓p-JNK	[168]
	NEAT1 ^a^	miR-204	↓Sorafenib-induced growth inhibition, ↑LC3-II/I ratio, ↑p-AKT, ↑p-mTOR, ↑ATG3	[169]
	DCST1-AS1 ^b^	-	↓Proliferation, ↓Migration, ↑Autophagy, ↑Apoptosis	[171]
	HAGLROS ^b^	miR-5095	↓LC3 II/I ratio, ↓Beclin-1, ↑p62, ↑Bax, ↑cle-Caspase 3, ↑cle-Caspase 9, ↓Bcl-2, ↓p-PI3K, ↓p-AKT, ↓p-mTOR, ↑PTEN	[172]
	DANCR ^b^	miR-222-3p	↓Proliferation, ↓Autophagy	[173]
	HULC ^a^	miR-15a	↑Proliferation, ↑LC3 II/I ratio, ↑Sirt1, ↓PTEN, ↑JAK, ↑PKM2, ↑CDK2, ↑p-PI3K, ↑p-AKT, ↑p-mTOR, ↑Jun, ↑Survivin	[174]
	ATB ^a^	-	↑Proliferation, ↑LC3 II/I ratio, ↓p-YAP ↑ATG5	[175]
	CRNDE ^a^	miR-543	↑ATG4B, ↑LC3-II/I ratio, ↓p62	[103]
	RP11-295G20.2 ^a^	PTEN	↓LC3B, ↓PTEN, ↑p-AKT, ↓FOXO3a	[176]
	CCAT2 ^b^	miR-4496/ELAVL1	↓Migration, ↓Invasion, ↓LC3 II/I ratio, ↓Beclin-1, ↑p62	[177]
	HnRNPU-AS1 ^a^	miR-556-3p/miR-580-3p	↓Proliferation, ↓Migration, ↑Autophagy,	[178]
Hypoxic Tumor	LincRNA-p21 ^b^	-	↓Proliferation, ↑G2/M arrest of cell populations, ↓Migration, ↓HIF-1α, ↓LC3 II, ↑p62	[217]
Laryngeal Squamous Cell Carcinoma	H19 ^b^	miR-107	↓LC3 II/I ratio, ↓Beclin-1, ↑p62, ↓LAMP2, ↓Chemoresistance	[179]
Lung Cancer	MSTO2P ^b^	-	↓Proliferation, ↓Agt5, ↓LC-3II, ↓EZH2	[180]
	LCPAT1 ^b^	RCC2	↓Proliferation, ↓Migration, ↓Invasion, Autophagy halted after CSE/ PM2.5 exposure	[181]
	LINC00857 ^b^	YBX1	↓Proliferation, ↑LC3 II/I ratio, ↑cle-PARP, ↓YBX1, ↓p-MET, ↑p-AMPKa	[218]
	PANDAR ^a^	-	↓Proliferation, ↑Autophagy, ↑Beclin-1	[182]
	MITA1 ^a^	-	↓Apoptosis, ↑LC3 II/I ratio, ↑Beclin-1, ↓p62, ↑Gefitinib resistance	[110]
Lymphoma	BCYRN1 ^a^	-	↑Proliferation, ↑Bcl-2, ↑Cyclin D1, ↓p53, ↓Bax, ↓p21, ↑Autophagy, ↑Beclin-1, ↑LC3-II, ↓p-mTOR, ↓p-AKT	[183]
Multiple Myeloma	MALAT1 ^b^	HMGB1	↓Proliferation, ↑Apoptosis, ↓Beclin-1, ↓LC3B, ↓HMBG1	[106]
Nasopharyngeal Cancer	MEG3 ^a^	miR-21	↑LC3 II/I ratio, ↑Beclin-1, ↓p62, ↑Bax, ↑cle-Caspase 3, ↓Bcl-2, ↑PTEN	[184]
Neuroblastoma	SNHG7 ^b^	miR-329-3p	↓Proliferation, ↓LC3B-I/LC3B-II, ↓Beclin-1, ↑p62, ↓Chemoresistance,	[219]
Non-Small Cell Lung Cancer	UCA1 ^b^	miR-185-5p	↓Proliferation, ↓Ki67, ↑Caspase 3, ↓LC3 II/I ratio, ↓Beclin-1, ↑p62, ↓WISP2, ↓β-catenin, ↓TCF4	[220]
	NBAT1 ^b^	PSMD10	↑LC3-II, ↓p62, ↑ATG7, ↑PSMD10, ↑Proliferation, ↑Chemoresistance	[185]
	BLACAT1 ^a^	miR-17	↑LC3 II/I ratio, ↑Beclin-1, ↑MRP1, ↑Chemoresistance, ↑Proliferation ↑ATG7	[186]
	GAS5 ^a^	-	↓Proliferation, ↑LC3-II ↓Chemoresistance	[187]
	PVT1 ^b^	miR-216b	↓LC3B II/I, ↑p62, ↓Beclin-1, ↑Apoptosis, ↑Cisplatin sensitivity,	[188]
Osteosarcoma	CTA ^a^	miR-210	↓LC3-II, ↓BNIP3/BNIP3L, ↑cle-Caspase 3, ↑Doxorubicin sensitivity, ↑Apoptosis	[189]
	DICER1-AS1 ^b^	miR-30b	↓ATG5, ↓LC3-II, ↓Beclin-1, ↓Proliferation, ↓Migration, ↓Invasion	[107]
	SNHG15 ^b^	miR-141	↓LC3-II/LC3-I, ↓ATG5, ↑p62, ↓Proliferation, ↓Migration, ↓Invasion	[190]
	SNHG6 ^b^	miR-26a-5p	↓ULK1, ↑ATF3, ↑cle-Caspase 3, ↓Proliferation, ↓Migration, ↓Invasion	[191]
Ovarian Cancer	HOXA11-AS ^b^	-	↑LC3II/I ratio, ↑Beclin-1, ↓p62, ↓Migration, ↓Invasion, ↑Cisplatin sensitivity	[192]
	TUG1 ^b^	miR-29b-3p	↓Beclin-1, ↓LC3B II/I, ↑cle-Caspase 3, ↑cle-Caspase 7, ↓Proliferation, ↑Paclitaxel sensitivity	[193]
	XIST ^b^	miR-506-3p	↓LC3 II/I ratio, ↑p62, ↑Bax, ↓Bcl-2, ↓FOXP1, ↑Carboplatin sensitivity	[194]
Pancreatic Cancer	LINC01207 ^b^	miR-143-5p	↓AGR2, ↓Cell growth, ↑Apoptosis, ↑LC3II, ↑Beclin-1, ↓p62, ↓Bcl-2/Bax	[195]
	PVT1 ^b^	miR-619-5p	↓ATG14, ↓Pygo2, ↓Cyclin-D1, ↓c-Myc, ↓LC3-II, ↑p62, ↓Axin2, ↓Gemcitabine resistance	[196]
	MALAT1 ^b^	HuR	↓LC3B II/I, ↑p62, ↓LAMP-2, ↓MMP-3, ↓MUC4	[197]
	SNHG14 ^b^	miR-101	↓RAB5A, ↓ATG4D, ↓Gemcitabine resistance, ↓Migration, ↓Invasion	[198]
	ANRIL ^b^	miR-181a	↓LC3 II, ↑Beclin-1, ↓HMGB1, ↓Proliferation, ↓Snail, ↓Vimentin, ↑E- cadherin, ↓N-cadherin	[199]
Papillary Thyroid Cancer	RP11-476D10.1 ^b^	miR-138-5p	↓LRRK2, ↑Beclin1, ↑LC3B, ↑Bax ↓Bcl-2	[221]
	BANCR ^b^	-	↓LC3-II/LC3-I, ↑Apoptosis, ↑Cell population in the G1 phase	[200]
Prostate Cancer	HULC ^b^	-	↑p-Beclin-1, ↑Bax, ↑Caspase 3, ↓PCNA, ↓Cyclin D1, ↑LC3B-II, ↑Irradiation sensitivity	[222]
	SNHG1 ^b^	EZH2	↑LC3-II, ↑Beclin-1, ↓p62, ↓p-PI3K, ↓p-AKT, ↓p-mTOR, ↓p-p70S6K, ↓Wnt1, ↓β-catenin, ↓c-Myc, ↓Cyclin D1, ↓EZH2	[201]
	PRRT3-AS1 ^b^	PPARγ	↑LC3A, ↑LC3B, ↑Beclin-1, ↓p-S6K1, ↓NF-κB1, ↓COX2, ↓p-4EPB1, ↓PCNA, ↓Ki67, ↑PPARγ, ↑Bax, ↑cle-Caspase 3, ↓Bcl-2, ↓Migration, ↓Invasion	[104]
Retinoblastoma	MALAT1 ^b^	miR-124	↓LC3-II, ↓Beclin-1, ↑p62	[202]
Uveal melanoma	ZNNT1 ^a^	-	↑ATG12, ↓SQSTM1, ↓Tumor cell growth, ↓Migration, ↓Invasion	[223]

^a^: Overexpression and their functional effect; ^b^: knockdown and their functional effect.

### 3.5. Gastric Cancer

Gastric cancer (GC), also called stomach cancer, is the 5th most common cancer occurring worldwide, which primarily arises from the lining of the stomach [1,224]. Although early interventions have been successful in treating this malignancy, it still ranks as the 4th leading cause of cancer-related deaths worldwide [1,225,226]. Hence, it is necessary to identify plausible mechanisms and causative factors that could be developed into treatment regimens. There has been a plethora of studies indicating the functional role of various lncRNAs in modulating autophagy to promote or inhibit tumorigenesis in GC [143,144,145]. For example, Wu and his group explored the oncogenic role of lncRNA small nucleolar host gene 11 (SNHG11) in promoting GC. SNHG11 was observed to be upregulated in both tumor tissues and cell lines of GC and was correlated with poor clinical outcomes in patients. It was observed that inhibiting SNHG11 led to the abrogation of various hallmarks of gastric cancer, such as proliferation, migration, invasion, stemness, and EMT, by preventing autophagy. Moreover, the post-transcriptional expression of autophagy-related 12 (ATG12) and catenin beta 1 (CTNNB1) was regulated by SNHG11 through the modulation of miR-483-3p/miR-1276. Further, SNHG11 initiated the ubiquitination of GSK-3β by interacting with Cullin 4A (CUL4A) to promote the activation of Wnt/β-catenin signaling cascade [143]. In another study, lncRNA JPX transcript, XIST activator (JPX) was found to be upregulated while the miR-197 level was downregulated and was associated with poor overall survival in patients. Further, miR-197 was established as a direct target of JPX, and the knockdown of JPX resulted in decreased cell migration, invasion, and modulated autophagy by regulating CXCR6 and miR-197. Moreover, the overexpression of miR-197 reduced the expression of CXCR6, Beclin-1, and the ratio of LC3-II/LC3-I levels with an increase in p62 protein expression [144]. Another group sought to evaluate the underlying mechanism of cisplatin resistance in GC regulated by LINC01572. Expression analysis revealed LINC01572 was upregulated in cisplatin-resistant GC patient samples and resistant cell lines. It was found that LINC01572 acted as a sponge for miR-497-5p, and the overexpression of miR-497-5p led to the blockage of ATG14, an essential protein in the autophagic pathway leading to apoptosis in drug-resistant cell lines. These results were further validated in xenograft mouse models, where the downregulation of LINC01572 resulted in increased tumor volumes by inhibiting autophagy through the miR-497-5p/ATG14 axis [145]. Other studies have also corroborated the important role of lncRNAs in regulating autophagy in gastric cancer [146,147,148,149,150,151,152]. Taken together, lncRNAs have proven to be indispensable regulators of autophagy in gastric tumorigenesis.

### 3.6. Glioma

Glioma is a prevalent tumor of the primary central nervous system, and the prognosis for this disease is poor [227,228]. Recently, many studies have reported the potential of autophagic drugs to induce cell death in glioma [229,230]. Thus, modulators of the autophagic process in glioma hold immense prospects in the management of this disease. In line with this, recent investigations have gauged the mechanistic role of lncRNAs in autophagy in glioma [90,105,160]. A study on lncRNA MALAT1 showed that it is an important activator of autophagy and promotes glioma cell proliferation by sponging miR-101. Additionally, the knockdown of MALAT1 resulted in the downregulation of ATG4D, STMN1, and RAB5A expression in glioma cell lines [105]. Another study on lncRNA AC023115.3 demonstrated that it inhibits the chemoresistance of glioblastoma by significantly reducing autophagy and sponging miR-26a [90]. Moreover, a study by Huo and his group revealed that lncRNA GAS5 facilitates the sensitivity of glioma cells by suppressing excessive autophagy via activation of the mTOR signaling cascade [159]. In addition, research conducted by Zheng and colleagues showed that Linc-RA1 is an inhibitor of autophagy. Further, it also promotes radioresistance by preventing the H2Bub1/USP44 complex in glioma cell lines [160]. Thus, the above studies demonstrate the crucial role of various lncRNAs in regulating autophagy in glioma. 

### 3.7. Hepatocellular Cancer

Hepatocellular cancer (HCC) is the most common malignancy of the liver and is also the third largest cause of cancer-related deaths worldwide [1,231]. Autophagy, through its various molecular associations, plays a crucial role in the pathogenesis of HCC [232]. Recently, various studies have explored the role of lncRNAs in regulating autophagy in HCC [168,169,173,216,233]. For instance, in a study on lncRNA activated by transforming growth factor beta (ATB), it was found to promote autophagy by activating YAP and inducing ATG5 in HCC cells [175]. Moreover, experiments on lncRNA HOTAIR demonstrated that it induced the activation of autophagy in HCC. It was also found that the overexpression of HOTAIR leads to the upregulation of ATG3 and ATG7 expression in HCC cell lines [98]. Another study revealed that the knockdown of lncRNA H19 resulted in a decrease in autophagic markers, such as LC3-II and Beclin-1, leading to activation of the PI3K/AKT/mTOR pathway in HCC [170]. In addition, it was found that lncRNA SNHG11 promotes autophagy, apoptosis, and proliferation via the regulation of hsa-miR-184/AGO2 in HCC [165]. Additionally, research on lncRNA colon cancer-associated transcript-1 (CCAT1) showed that it aids I and promotes autophagy by functioning as a sponge for miR-181a-5p, thereby regulating the expression of ATG7 in HCC [167]. Further, lncRNA MEG3 was found to contribute towards the inhibition of autophagy by regulating the expression of Beclin-1 and PI3K/AKT/mTOR signaling pathways [215]. Another study discovered that the lncRNA hepatocyte nuclear factor 1α (HNF1A-AS1)/miR-30b-5p axis promotes autophagy, and ATG5 was identified as a target of miR-30b-5p [97]. Furthermore, lncRNA MCM3AP-AS1 was found to be involved in regulating autophagy and promoting HCC metastasis by interacting with miR-455 and regulating epidermal growth factor receptor (EGFR) [216]. Another study on lncRNA neighbor of BRCA1 lncRNA 2 (NBR2) revealed its tumor-suppressive role in HCC by inhibiting Beclin-1-dependent autophagy pathway via JNK and ERK signaling cascades [168]. In one study, Li and colleagues revealed that lncRNA DCST1 antisense RNA 1 (DCST1-AS1) is a crucial tumor-promoting factor in HCC. It was found to promote cell proliferation and inhibit autophagy and apoptosis via the AKT/mTOR signaling cascade [171]. Additionally, research on lncRNA nuclear-enriched abundant transcript 1 (NEAT1) demonstrated that it promotes autophagy via modulation of the miR-204/ATG3 pathway in HCC [169]. Further, Wei and colleagues elucidated the role of lncRNA HAGLR opposite strand (HAGLROS) in regulating autophagy in HCC. HAGLROS enhances autophagy and promotes cell proliferation by regulating the miR-5095/ATG12 axis [172]. Furthermore, a study on lncRNA highly upregulated in liver cancer (HULC) revealed that it inhibits PTEN via the ubiquitin-proteasome system mediated by the autophagy-related p62 protein, which leads to activation of the PI3K–AKT–mTOR pathway in HCC cells [174]. Further, a study on lncRNA DANCR showed that it promotes the expression of ATG7, which leads to autophagy and HCC cell proliferation by sponging miR-222-3p [173]. Taken together, the above studies elucidated the role of various lncRNAs in promoting autophagy in HCC.

### 3.8. Hematological Malignancies

Hematological malignancies are commonly called cancers of the blood and its associated tissues and cells, such as bone marrow and immune cells [234,235]. Though the conventional therapeutic approaches have increased patients’ overall survival and quality of life, the clinical outcomes suffer with drug tolerance and tumor reoccurrence [236]. There is heterogeneity among the various subclasses of blood cancers, which can be stratified into lymphoma, leukemia, and myeloma [237]. Recent studies have delineated the functional role of different lncRNAs in modulating autophagy in various hematological malignancies [125,126,212]. For instance, a study by Zhang et al. elucidated the functional role of lncRNA LINC00265 in acute myeloid leukemia (AML). It was observed that LINC00265 was upregulated and miR-485-5p was significantly downregulated in AML patient serum and cell lines. Moreover, the overexpression of LINC00265 was found to promote autophagy and suppress apoptosis in AML cell lines by upregulating LC3-II/LC3-I and Beclin expression while decreasing p62 levels. In addition, LINC00265 showed a direct interaction with miR-485-5p, where lncRNA served as a ceRNA to regulate the expression of IRF2 [125]. Another study explored the oncogenic potential of lncRNA UCA1 in modulating AML proliferation and autophagy. It was reported that UCA1 acted as a sponge to bind miR-96-5p, which in turn induced the ATG7/autophagic pathway in AML cells. Moreover, UCA1 was found to promote the proliferation of AML cells through the activation of autophagy [212]. Additionally, Zhang and his group studied the tumor-promoting and drug resistance role of lncRNA DANCR in AML. The overexpression of DANCR resulted in an increase in cytarabine resistance in AML. Further, DANCR induces autophagy by sponging the miR-20a-5p levels and increasing the expression of ATG16L1 [126]. Thus, these studies have proved the potential of autophagic modulators, such as lncRNAs, as a treatment approach in different hematological malignancies.

### 3.9. Lung Cancer

Lung cancer is one of the most frequent malignancies worldwide due to various risk factors, such as smoking, drinking, and air pollution, etc. [1,238,239,240,241,242]. Various studies have found that lncRNAs play a vital role in the progression of lung cancer [243,244,245]. With regard to this, a study on lncRNA misato homolog 2 pseudogene (MSTO2P) showed that it was considerably upregulated in lung cancer cells. The knockdown of MSTO2P resulted in decreased levels of LC3-I, LC3-II, AGT5, and EZH2, leading to impaired processes of proliferation and autophagy in lung cancer cells [180]. An analysis by Len Zhang and his group suggested that lncRNA promoter of CDKN1A antisense DNA damage activated RNA (PANDAR) inhibited the proliferation of NSCLC cells via the activation of autophagy, as well as apoptotic pathways, by upregulating the expression of BECN1 [182]. Moreover, another study indicated that lncRNA lung cancer progression-association transcript 1 (LCPAT1) was expressed in the presence of particulate matter (PM) 2.5 and cigarette smoke extracts. It stimulated the progression of lung cancer through RCC2 leading to the upregulation of autophagy [181]. Another study found that LINC00857 knockdown induced autophagy by increasing the phospho-AMP-activated protein kinase (p-AMPK). Thus, it was found that LINC00857/p-AMPKa signaling is vital for regulating autophagy, apoptosis, and cell proliferation. This could lead to a potential therapeutic target for lung cancer [218]. In addition, it was observed that the UCA1 inhibitory effect on miR-185-5p was significantly decreased after interference with lncRNA UCA1. This resulted in the downregulation of Beclin-1, β-catenin/TCF-4, and LC3II, leading to a reduction in the growth of cells and autophagy in non-small cell lung cancer (NSCLC) [220]. Apart from this, lncRNA neuroblastoma-associated transcript 1 (NBAT1) was also found as an autophagy inhibitor by suppressing ATG7 in NSCLC [185]. Additionally, lncRNA bladder cancer-associated transcript 1 (BLACAT1) was found to be upregulated in NSCLC. It promoted chemoresistance and autophagy of the cancer cells via the miR-17/ATG7 axis [186]. Further, a study suggested that lncRNA plasmacytoma variant translocation 1 (PVT1) might function as a ceRNA for miR-216b. It inhibited the cisplatin sensitivity of NSCLC by regulating autophagy and apoptosis. This might provide a novel target for improving the efficiency of chemotherapy in NSCLC [188]. Furthermore, one study showed that the downregulation of lncRNA GAS5 was related to cisplatin resistance in NSCLC. The knockdown of GAS5 decreased autophagy and promoted cisplatin resistance in NSCLC [187]. Thus, these studies demonstrate the importance and potential of lncRNAs in the treatment of lung cancer by modulating the autophagic pathways.

### 3.10. Osteosarcoma

Osteosarcoma is the most common type of bone cancer, which originates in the mesenchymal tissue and affects children and young adults worldwide [246]. A growing line of experimental evidence suggests that lncRNAs play a vital role in osteosarcoma [247,248,249]. In accordance with this, Wang and his group aimed to explore the functional role of lncRNA CTA in osteosarcoma chemoresistance. lncRNA CTA was found to be downregulated in osteosarcoma tissues as compared to levels i the normal adjacent tissues. It was observed that the overexpression of lncRNA CTA resulted in decreased cell proliferation with an increase in autophagy and DOX-induced apoptosis in MG63 and Saos-2 osteosarcoma cell lines. Treatment with DOX increased the accumulation of LC3B-II and BNIP3/BNIP3L levels, whereas the overexpression of lncRNA CTA reversed this effect [189]. Another study reported that lncRNA DICER1 antisense RNA 1 (DICER1-AS1) enhanced the proliferation and autophagy of osteosarcoma cells by regulating the miR-30b/ATG5 levels. The knockdown of DICER1-AS1 led to decreased proliferation, migration, and invasion of osteosarcoma cells. Further, ATG5 was found to be a target of miR-30b, which was regulated by lncRNA DICER1-AS1 [107]. Moreover, in another study, lncRNA SNHG15 was found to be negatively correlated with the miR-141 levels in osteosarcoma. The knockdown of SNHG15 abrogated LC3B-II expression while increasing p62 levels, and the introduction of miR-141 rescued autophagy-related protein expression [190]. In addition, Zhu et al. demonstrated the tumorigenic potential of lncRNA SNHG6 in osteosarcoma. SNHG6 was found to be overexpressed in osteosarcoma cells and tissues, and its high expression was correlated with poor survival and metastasis. Moreover, SNHG6 silencing decreased proliferation, migration, and autophagy and induced G0/G1 phase arrest and apoptosis in osteosarcoma [191]. Overall, these studies delineate the crucial role of lncRNAs in autophagy and could be used as a potential target in the treatment of osteosarcoma.

### 3.11. Ovarian Cancer

Ovarian cancer is the most common gynecological cancer afflicting women worldwide [1,250]. Although treatment options, such as surgical resection and chemotherapy, have increased ovarian cancer patients’ survival, tumor relapse and chemoresistance are the major hurdles in efficacious treatments [251]. Studies have identified lncRNAs as playing a crucial role in modulating autophagy in ovarian cancer. For example, Chen and co-workers explored the role of lncRNA HOXA11-AS in the development of ovarian cancer. It was observed that HOXA11-AS was overexpressed in ovarian cancer, and the knockdown of HOXA11-AS resulted in the inhibition of proliferation and malignant transformation with the induction of cell cycle arrest, apoptosis, and autophagy in ovarian cancer. The silencing of this lncRNA was also found to elevate autophagy-related proteins, such as Beclin-1, and increase the LC3II/I ratio while reducing p62 protein expression [192]. Moreover, in another study, lncRNA TUG1 was found to be overexpressed in ovarian cancer tissue samples and cell lines. Silencing TUG1 decreased Beclin-1 and the conversion of LC3B-I to LC3B-II in ovarian cancer cells. Further, it was observed that the knockdown of TUG1 affected paclitaxel sensitivity in cancer cells by modulating autophagy through the sponging of miR-29b-3p levels [193]. In addition, Xia et al. deciphered the mechanistic role of lncRNA X-inactive specific transcript (XIST) in carboplatin resistance in ovarian cancer. lncRNA XIST was found to be upregulated in ovarian cancer and carboplatin-resistant cells. Moreover, the knockdown of XIST led to the suppression of proliferation and autophagy while inducing apoptosis in ovarian cancer cells. Further, miR-506-3p was found to be a target for XIST, and it mediated carboplatin resistance by modulating FOXP1 expression [194]. Taken together, lncRNAs have been found to modulate autophagy and chemotherapy resistance; however, further studies are required to decipher the better functional role of lncRNAs that modulate autophagy in ovarian cancer.

### 3.12. Pancreatic Cancer

Pancreatic cancer is one of the major causes of cancer-related deaths worldwide due to its poor prognosis and few therapeutic approaches at the advanced stages [252,253]. Therefore, understanding the molecular mechanism and the causative factors involved in this deadly malignancy would open newer possibilities for novel diagnostic, prognostic, and therapeutic interventions. Various studies have delineated the involvement of lncRNAs as crucial regulators in pancreatic cancer [42,254,255]. For example, the role of lncRNA LINC01207 was evaluated in modulating autophagy and apoptosis in pancreatic cancer. It was found that lncRNA LINC01207 was upregulated in pancreatic cancer, and silencing LINC01207 increased Beclin-1 and LC3II expression while decreasing the p62 levels and Bcl-2/Bax ratio. Moreover, LINC01207 was reported to modulate the expression of AGR2 by directly targeting miR-143-5p [195]. In addition, lncRNA PVT1 was observed to promote autophagy and gemcitabine resistance in pancreatic cancer by regulating miR-619-5p/ATG14 via the Wnt/β-catenin pathway. It was also found that the interaction of PVT1 and ATG14 facilitates the formation of the autophagy-specific complex I (PtdIns3K-C1) and class III PtdIns3K activity, thereby initiating autophagy in pancreatic cancer [196]. MALAT1 is a well-researched lncRNA known for its oncogenic role and for regulating the process of autophagy in different cancers, including pancreatic malignancy. For instance, MALAT1 was found to be positively correlated with LC3B expression. Further, silencing MALAT1 abrogated the autophagic flux by modulating LC3, P62, and LAMP-2. It was also speculated that MALAT1 would regulate autophagic processes by interacting with HuR via the regulation of TIA-1 (T-cell intracellular antigen-1) levels [197]. In addition, the lncRNA SNHG14 was found to enhance gemcitabine resistance and induce autophagy in pancreatic cancer by sponging its target, miR-101. It was also found that silencing SNHG14 markedly reduced autophagy-related protein 4D (ATG4D) and RAB GTPase 5 A (RAB5A) in SW1990 pancreatic ductal adenocarcinoma cells [198]. Further, Wang and his group investigated the chemoresistance effects of lncRNA antisense non-coding RNA in terms of the INK4 locus (ANRIL) in pancreatic cancer. It was found that ANRIL was overexpressed in pancreatic tissues while miR-181a was inversely downregulated. The same study also showed that ANRIL-induced HMGB1 mediated autophagy by targeting miR-181a [199]. Hence, a vivid understanding of the mechanistic role of lncRNAs in autophagy would be crucial in designing novel therapeutic regimens against pancreatic cancer.

### 3.13. Prostate Cancer

Prostate cancer is one of the most commonly diagnosed cancers in men worldwide [256,257]. According to GLOBOCAN 2020, approximately 1,414,259 new incidences were reported for prostate cancer [1]. Although there have been numerous reports of lncRNAs regulating prostate tumorigenesis, very few studies have shed light on the regulation of autophagy by lncRNAs in prostate cancer [104,201,222]. In line with this, Chen and his colleagues explored the mechanistic role of lncRNA HULC in prostate cancer treatment via irradiation. The knockdown of HULC resulted in a significant decrease in cellular proliferation and the induction of apoptosis in PC3 and LNCaP cells treated with irradiation. In addition, HULC inhibited autophagy by regulating the levels of Beclin-1 and targeting the mTOR pathway [222]. In addition, lncRNA SNHG1 was found to inhibit autophagy by binding to EZH2 and modulating the PI3K/AKT/mTOR and Wnt/β-Catenin pathways. Further, interference with SNHG1 resulted in the inhibition of proliferation, migration, and invasion in LNCaP and PC3 prostate cancer cells [201]. Further, LncRNA PRRT3-AS1 was found to be highly expressed in prostate cancer. Therefore, the knockdown of PRRT3-AS1 induced autophagy by decreasing COX2, S6K1, NF-κB1, and 4EPB1 and upregulating peroxisome proliferator-activated receptor γ (PPARγ), thereby inhibiting the mTOR pathway. Furthermore, PPARγ was found to be the direct target of lncRNA PRRT3-AS1 [104]. Hence, these studies clearly depict the vital role of lncRNA in regulating autophagy and suggest possible therapeutic interventions.

### 3.14. Other Cancers

Besides the aforementioned studies, lncRNAs have been found to play a crucial role in regulating autophagy in different cancers of the eye and thyroid [200,202,221,223]. Uveal melanoma (UM), a type of rare cancer, occurs in the melanocytes present in the uveal tract of the eyes [258]. LncRNA was also found to modulate autophagy in this cancer. For example, Li P and his group showed that the overexpression of lncRNA ZNNT1 induced autophagy by increasing the levels of ATG12 and the degradation of SQSTM1, which led to the suppression of growth and migration in OCM1 and OM431 uveal melanoma cell lines. Further, the knockdown of ZNNT1 resulted in the repression of PP242-induced autophagy [223]. Retinoblastoma, another type of eye cancer, starts at the retina (back of the eyes) and is usually common in children [259]. The modulatory effect of lncRNAs was also reported in retinoblastoma. With regard to this, one study elucidated the functional role of lncRNA MALAT1, a well-known oncogenic lncRNA, in retinoblastoma. It was found that MALAT1, through the direct targeting miR-124, modulated the expression of Syntaxin 17 (STX17), one of the SNARE proteins of the autophagosome [202].

There have been few reports where lncRNAs were found to be involved in autophagic regulation in papillary thyroid cancer [200,221]. In line with this, one study found that lncRNA RP11-476D10.1 was overexpressed in papillary thyroid cancer cells. Further, the knockdown of RP11-476D10.1 resulted in a decrease and increase in apoptosis and autophagy, respectively. Furthermore, miR-138-5p was found to be a direct target of RP11-476D10.1, where it activated the expression of LRRK2 [221]. Another study found that BRAF-activated lncRNA (BANCR) promoted papillary thyroid carcinoma by inducing proliferation and autophagy. The overexpression of BANCR led to upregulation of the LC3-II/LC3-I ratio, a marker for autophagy [200]. Taken together, lncRNAs are known to modulate the expression of autophagy-related genes by acting as ceRNAs, and targeting these lncRNAs might be a plausible approach on the path of cancer treatment.

## 4. Conclusions and Future Prospects

Cancer has become the most prevalent disorders of this century, accounting for millions of deaths worldwide. Though the current treatment approaches, such as surgical resection, chemotherapy, radiotherapy, and targeted therapies, have increased the overall survival of patients and the quality of life, there is no definite cure for the advanced stages of the diseases. Moreover, most treatment regimens suffer from poor clinical outcomes with severe side effects. Hence, there exists a need to understand the intricate molecular events underlying tumorigenesis in order to develop safe and targeted therapies. With the discovery of autophagy, a crucial cellular mechanism governing homeostasis, there has been huge attention towards understanding its mechanistic role in health and diseases. Autophagy, a catabolic degradative process, helps in the removal of unwanted protein aggregates and damaged organelles and thus maintains nutrient recycling under stress stimuli. A plethora of studies has demystified the crucial role of autophagy in various chronic diseases, including cancer. In cancer, autophagy plays a dual role; it acts as a tumor suppressor to degrade the damaged and accumulated proteins, thereby reducing cancer development, but in advanced stages of the disease, it sustains cancer cell proliferation and viability by providing energy during nutritional starvation. In some cases, autophagy may prevent the development of tumors, but mounting evidence points to this pathway’s pro-tumorigenic effects. In this context, autophagy causes tumor-intrinsic and tumor-extrinsic immunosuppression, metabolic alterations, and resistance to treatment. Various studies have shown that lncRNAs are essential for the initiation and development of different types of cancer. In particular, aberrant lncRNA expression may be present in cancer cells with DNA damage, immunological evasion, and cellular metabolic problems. The complex process of carcinogenesis is even more intriguing because of the diversity and heterogeneity of lncRNAs. Creative experimentation and advanced next-generation technologies have widened our understanding of lncRNAs’ role in cancer. Because the sensitivity and specificity are still not at the acceptable level, it is challenging to use lncRNAs as cancer biomarkers at present. However, developing diagnostics and tailored cancer therapies might be possible using the autophagy-modulating lncRNAs. We may conclude from the rising body of evidence on lncRNAs, autophagy, and malignancies that the majority of lncRNAs have a role in carcinogenesis by stimulating or inhibiting the autophagy process. In this review, we have discussed lncRNAs that modulate autophagy to either promote or inhibit different types of cancer. LncRNAs, members of various ceRNA types, employ the ceRNA network’s mechanism to regulate epigenetic control and vital post-transcriptional regulation. These days, mounting data show that intracellular lncRNA availability is sufficient in disease states, such as neoplasms, to induce ceRNA crosstalk of the lncRNA/miRNA/mRNA axis. Additionally, lncRNA could sponge miRNA for an extended period through incomplete complementary binding between miRNA-responsive elements and miRNA, changing the activity and availability of miRNA while regulating the expression of autophagy-related genes. We have also shed light on the mechanistic effects of the role of lncRNA based on the ceRNA network and how autophagic modulation brings about a change in different hallmarks of cancer. So far, all of the studies have been inclined towards the modulation of autophagy by lncRNAs. It would be a crucial question to ask whether autophagy can regulate the expression of lncRNA. To date, only PVT1 expression has been found to be modulated with autophagy in diabetes.

Since their discovery, lncRNAs that modulate autophagy have been the subject of intense debate. Both autophagy and lncRNAs have the potential to either promote or inhibit the development of cancer. While increasing autophagy is a useful therapeutic approach for treating chronic inflammatory disorders, autoimmune disease, and neuroinflammation, its significance in cancer has remained pleiotropic. Sustained autophagy is recognized as a crucial mechanism for treating resistance and immune evasion in the context of cancer. Pharmacological strategies that safely reduce autophagic flux to support meaningful immune responses against advanced stages of cancer while simultaneously allowing for the emergence of long-lasting protection as determined by antigen-specific cellular immunity will be necessary for the modulation of autophagy to be successful. The conception to use non-coding RNA as a therapeutic regimen has gained immense attention from researchers worldwide. Many antisense oligonucleotides and small interfering RNAs have been used in the clinical use of RNA-based treatments against various diseases over the past ten years, and several of these have been approved by the Food and Drug Administration. The clinical trial results, however, have thus far been mixed, with some studies reporting strong potent effects and others showing little efficacy or toxicity. One of the biggest hurdles in employing lncRNAs is the efficient distribution of RNA across the cell membrane and reaching the site and cell type of interest to carry out their post-transcriptional regulation. Innovative technologies are coming up front with an unprecedented interdisciplinary approach that could provide plausible solutions to these problems. Still, there is a long way ahead for the lncRNAs to enter clinical trials. What we require now is more experimental evidence of the lncRNA regulation of autophagy in different pre-clinical cancer models. Further, randomized multi-centered clinical trials are paramount in establishing lncRNAs as a vital therapeutic approach targeting autophagy in cancers.

## Data Availability

Not applicable.

## Abbreviations

5-FU: 5-flurouracil; ADAMTS9-AS1: ADAM metallopeptidase with thrombospondin type 1 motif, 9 (ADAMTS9); antisense RNA 1; AML: acute myeloid leukemia; AMPK: adenine monophosphate-activated protein kinase; ANRIL: antisense non-coding RNA in the INK4 locus; ATB: activated by transforming growth factor beta; ATG: autophagy-related genes; ATRA: all-trans retinoic acid; BANCR: BRAF-activated lncRNA; Bcl-2: B-cell lymphoma 2; BLACAT1: bladder cancer associated transcript 1; CASC2: cancer susceptibility candidate 2; CCAT1: colon cancer-associated transcript-1; ceRNA: competing endogenous RNAs; Chast: cardiac hypertrophy-associated transcript; CMA: chaperon-mediated autophagy; CPS1-IT1: CPS1 intronic transcript 1; CRC: colorectal cancer; CRNDE: Colorectal neoplasia differentially expressed; CUL4A: Cullin 4A; DANCR: differentiation antagonizing non-protein coding RNA; DCST1-AS1: DCST1 antisense RNA 1; DDP: Cisplatin; DFCP1: double FYVE-containing protein 1; DICER-AS1: DICER1 antisense RNA 1; EGFR: epidermal growth factor receptor; EGOT: eosinophil granule ontogeny transcript; EMT: epithelial-to-mesenchymal transition; GAS5: growth arrest-specific transcript 5; GC: gastric cancer; GSK3: glycogen synthase kinase-3; H19: H19 imprinted maternally expressed transcript; HAGLROS: HAGLR opposite strand; HCC: hepatocellular cancer; HIF-1α: hypoxia-inducible factor 1-alpha; HNF1A-AS1: hepatocyte nuclear factor 1α; HOTAIR: HOX antisense intergenic RNA; HOTAIRM1: HOXA transcript antisense RNA myeloid-specific 1; HULC: highly upregulated in liver cancer; JPX: JPX transcript, XIST activator; KCNQ1OT1: KCNQ1 opposite strand/antisense transcript 1; LCPAT1: lung cancer progression-association transcript 1; Linc-ROR: lincRNA, regulator of reprogramming; lncRNA: long non-coding RNA; MALAT1: metastasis-associated lung adenocarcinoma transcript 1; MEG3: maternally expressed gene 3; miR: microRNA; mTORC1: mammalian target of rapamycin complex 1; MTSO2P: misato homolog 2 pseudogene; NBAT1: neuroblastoma associated transcript 1; NBR2: neighbor of BRCA1 gene 2; ncRNA: non-coding RNA; NEAT1: nuclear paraspeckle assembly transcript 1; NSCLC: non-small cell lung cancer; PANDAR: promoter of CDKN1A antisense DNA damage activated RNA; PAS: pre-autophagosomal structure; PI3K: phosphoinositide 3-kinase; PI3P: phosphatidylinositol 3-phosphate; Plekhm1: pleckstrin homology domain-containing protein family M member 1; PPARγ: peroxisome proliferator-activated receptor γ; PTENP1: PTEN pseudogene-1; PVT1: plasmacytoma variant translocation 1; ROR1-AS1: ROR1 antisense RNA 1; ROS: reactive oxygen species; SLCO4A1-AS1: SLCO4A1 antisense RNA 1; SNGH14: small nucleolar RNA host gene 14; SNHG11: small nucleolar host gene 11; SNHG6: small nucleolar RNA host gene 6; SQSTM1: sequestosome 1; TRIM16: tripartite motif-containing 16; TUG1: taurine-upregulated gene 1; UCA1: urothelial carcinoma-associated 1; ULK1: autophagy activating kinase 1.
